# Health impact of the Tajogaite volcano eruption in La Palma population (ISVOLCAN study): rationale, design, and preliminary results from the first 1002 participants

**DOI:** 10.1186/s12940-024-01056-4

**Published:** 2024-02-13

**Authors:** María Cristo Rodríguez-Pérez, Manuel Enrique Fuentes Ferrer, Luis D. Boada, Ana Delia Afonso Pérez, María Carmen Daranas Aguilar, Jose Francisco Ferraz Jerónimo, Ignacio García Talavera, Luis Vizcaíno Gangotena, Arturo Hardisson de la Torre, Katherine Simbaña-Rivera, Antonio Cabrera de León

**Affiliations:** 1 University Hospital Nuestra Señora de Candelaria and Primary Care Authority of Tenerife, Santa Cruz de Tenerife, Spain; 2 Toxicology Unit, Research Institute of Biomedical and Health Sciences (IUIBS), University of Las Palmas de Gran Canaria (ULPGC), Las Palmas de Gran Canaria, Spain; 3 Spanish Biomedical Research Centre in Physiopathology of Obesity and Nutrition (CIBERObn), Madrid, Spain; 4 Primary care health centre of Breña Alta. Health Services Authority of La Palma, Breña Alta, Spain; 5 University hospital of La Palma. Health Services Authority of La Palma, Breña Alta, Spain; 6 Primary care health centre of Breña Baja. Health Services Authority of La Palma, Breña Alta, Spain; 7 Respiratory Department, University Hospital Nuestra Señora de Candelaria., Santa Cruz de Tenerife, Spain; 8 Toxicology Department, Medical School, University of La Laguna, San Cristóbal de La Laguna, Spain; 9 Centro de Investigación para la Salud en América Latina (CISeAL), Facultad de Medicina, Pontificia Universidad Católica del Ecuador (PUCE), Quito, Ecuador; 10 Preventive Medicine Department, Medical School, University of La Laguna, San Cristóbal de La Laguna, Spain

**Keywords:** Volcanic eruptions, Epidemiology and Public Health, Morbidity Associated with volcanic eruption, Mortality Associated with volcanic eruption, Non-anthropogenic toxic contaminants

## Abstract

**Background:**

The eruption of the Tajogaite volcano began on the island of La Palma on September 19, 2021, lasting for 85 days. This study aims to present the design and methodology of the ISVOLCAN (Health Impact on the Population of La Palma due to the Volcanic Eruption) cohort, as well as the preliminary findings from the first 1002 enrolled participants.

**Methods:**

A prospective cohort study was conducted with random selection of adult participants from the general population, with an estimated sample size of 2600 individuals. The results of the first 857 participants are presented, along with a group of 145 voluntary participants who served as interveners during the eruption. Data on epidemiology and volcano exposure were collected, and participants underwent physical examinations, including anthropometry, blood pressure measurement, spirometry, and venous blood extraction for toxicological assessment.

**Results:**

In the general population (*n* = 857), descriptive analysis revealed that the participants were mostly middle-aged individuals (50.8 ± 16.4), with a predominance of females. Before the eruption, the participants resided at a median distance of 6.7 km from the volcano in the Western region and 10.9 km in the Eastern region. Approximately 15.4% of the sample required evacuation, whose 34.8% returning to their homes on average after 3 months. A significant number of participants reported engaging in daily tasks involving cleaning of volcanic ash both indoors and outdoors. The most reported acute symptoms included ocular irritation, insomnia, mood disorders (anxiety-depression), and respiratory symptoms. Multivariate analysis results show that participants in the western region had a higher likelihood of lower respiratory tract symptoms (OR 1.99; 95% CI:1.33–2.99), depression and anxiety (OR 1.95; 95% CI:1.30–2.93), and insomnia (OR 2.03; 95% CI:1.33–3.09), compared to those in the eastern region.

**Conclusion:**

The ongoing follow-up of the ISVOLCAN cohort will provide valuable insights into the short, medium, and long-term health impact related to the material emitted during the Tajogaite eruption, based on the level of exposure suffered by the affected population.

**Supplementary Information:**

The online version contains supplementary material available at 10.1186/s12940-024-01056-4.

## Background

Approximately one billion people worldwide live within the influence zone of an active volcano, at about 100 km [1], and thus could be affected by the effects of an eruption at some point. On La Palma Island (Canary Islands, Spain), a volcanic eruption began on September 19, 2021, in the Valle de Aridane, lasting for 85 days and resulting in the formation of a new volcano named Tajogaite. The eruption generated a significant expulsion of volcanic ash and gas emissions, leading to days of highly unfavourable air quality with elevated toxicity levels in the breathable air [2].

Volcanic eruptions can have a wide range of deleterious effects on human health. Despite their often-short duration, the emission of toxic gases, particles, and ash deposits can persist in the local environment for years or even decades, being mobilized and redistributed by climatic factors or human activities [3]. Gases emitted during volcanic activity, such as CO and CO_2_, SO_2_, HCl, HF, H_2_S, radon, and permanent degassing, have the potential to impact human health [4]. There are several causes for degassing, both natural and anthropogenic sources whose contribute to air pollution. In La Palma, several years before the Tajogaite eruption, the concentration of CO_2_ were recorded in Cumbre Vieja, beeing related, in much more amount, to anthropogenic and other natural sources than magmatic emissions, accounting for only 4% [5]. Acute and prolongated exposure of individuals to high concentrations of CO_2_ is uncommon and incompatible with life. Nevertheless, long-term exposure occurs at low concentrations and outdoor is more frequent. In this context, physiological adaptation mechanisms are activated, promote oxidation followed by the release of proinflammatory cytokines; these mechanisms entail the development of a pro-inflammatory status and, consequently, the onset of diseases related to such conditions [6]. Particularly harmful to health is the atmospheric transformation of SO_2_ and other gases into particulate matter (PM) [7]. This transformation process is influenced by various factors, including the emission plume’s characteristics and maturity, as well as meteorological variables such as humidity, solar radiation, and temperature [7].

Previous studies have demonstrated an increase in acute symptoms of ocular irritation and upper respiratory tract issues [8], as well as elevated respiratory morbidity and visits to hospital or primary care services due to exacerbation of respiratory pathologies associated with peaks in airborne emissions of these types of toxic gases or particles [3, 9, 10]. Many of these associations are independent of age, sex, education level, and smoking habits, exhibiting a dose-response gradient [11]. Furthermore, exposure has been linked to increased cardiovascular morbidity and all-cause mortality [12]. Few studies have assessed long-term chronic health effects, and the scarce longitudinal studies have methodological limitations due to the analysis of samples from hospitalized patients, low reliability of data sources in some countries, and short follow-up periods, often not exceeding six months. Consequently, longitudinal studies with extended follow-up periods in the general population are needed to analyse the occurrence of deleterious medium to long-term effects.

In January 2022, the ISVOLCAN study (Health Impact on the Population of La Palma caused by the Tajogaite Volcano Eruption) was started. This study involves the recruitment and follow-up of a cohort from the general adult population to assess the impact of the Tajogaite volcano eruption on the health of the population of the island. The purpose of this paper is to present the methodology of ISVOLCAN study and provide a preliminary analysis of the data obtained from the first 1002 enrolled participants.

## Methods

### Study design

This is an observational epidemiological study, using a prospective cohort design, targeting the general adult population residing in multiple municipalities on La Palma Island. Additionally, a group of volunteers from professional personnel with access to the exclusion zone or operations centre during the eruption (including civil protection workers, Spanish Security Forces, Emergency Services, scientists, etc.) was included. The study consists of two different stages: the first stage involved recruitment and baseline assessments conducted from 2022 to 2023, while the second phase will involve follow-up of the cohort at 2, 5, and 10-year intervals.

The study has obtained authorization from the health authorities and received a favorable decision from the Provincial Ethics and Medicines Committee (ref. CHUNSC_2021_88). Participants were required to provide written consent before being included in the study.

### Settings

La Palma Island is a volcanic island located in the Atlantic Ocean, within the Canary Islands archipelago, Spain. Geographically, it is positioned at 28° 26’ N latitude and 14° 01’ W longitude from Madrid. Covering an approximate area of just over 700 km^2^, it ranks as the fifth-largest island in this archipelago [13]. The island counts with a population of 83,439 inhabitants [14]. Los Llanos de Aridane in the west side, is the city with highest population density, followed of Santa Cruz de La Palma. The Canarian Public Health System provides healthcare to the entire population through a hospital and a network of primary care health centres throughout the island.

On September 19, 2021, the eruption started in the western region of La Palma Island, in Cabeza de Vaca area, on the western flank of the Cumbre Vieja ridge, belowing to the municipality of El Paso. This volcanic process persisted for 85 days until its conclusion on December 13 of the same year. It consisted in a long-lasting, hybrid eruption associated with multiple eruptive styles (effusive, lava fountains, ash emissions, strombolian explosions) with the formation of cones of various heights, widespread tephra blankets and extensive lava-flow fields and was characterized by simultaneous effusive and explosive activity [15]. The eruption affected the Valle de Aridane, which was greatly impacted by the lava flows, gases, and particulate matter emitted during the eruption. The newly formed volcano, named Tajogaite, reached a maximum altitude of 1131 m above sea level and extended 200 m from the pre-eruptive topography, with its base situated at 1080 m above sea level [16].

### Subjects, sampling, inclusion and exclusion criteria

#### Participants from the general population

The sample selection was conducted using a random, stratified approach based on age and gender groups, according to the 2020 municipal census data of the population residing in the western region (El Paso, Los Llanos, Tazacorte, and Puntagorda) and eastern region (Mazo, Santa Cruz de La Palma, and San Andrés y Sauces) of the island.

The sample was drawn from the health card registry of the Canarian Health Service, which is continuously updated and includes all individuals above the age of 18 who were residents on the island during the eruption and provided informed consent to participate in the study.

To ensure the achievement of the intended objectives, the population of the western region, closer to the eruption, was oversampled. The sample sizes for each municipality in the western region were as follows: Los Llanos de Aridane: 820; El Paso: 405; Tazacorte: 305; Puntagorda: 205. In contrast, for the eastern region, the sample sizes were 505 for Santa Cruz de La Palma, 205 for Mazo, and 155 for San Andrés y Sauces.

### Highly exposed participants (intervening personnel)

Using a non-probabilistic convenience sampling method, participants in the study also included members of various professional and volunteer groups involved in different tasks related to the eruption and who had access to the volcano’s exclusion zone or operations centre. Although access to these areas was controlled and followed safety and protection measures, we expected that these participants from the different groups were highly exposed during their workdays throughout the nearly four-month duration of the eruption.

### Study size

An initial sample size of 1207 persons was estimated (precision 3%, confidence level 95%) based on an expected prevalence of acute respiratory symptoms (the most frequently associated with such phenomena). Considering an anticipated participation rate in this type of study of 60–70% and a dropout rate during follow-up exceeding 30%, the sample was increased to 2600 individuals.

### Recruitment and baseline assessment

In January 2022, telephone contact with the selected sample started. Those who agreed to participate in the study were administered an epidemiological questionnaire specifically designed for this purpose. The questionnaire was completed by Primary Care professionals, including both physicians and nurses, who were trained for this study. Subsequently, participants were scheduled to visit the health centres in the two regions for physical examinations, pulmonary function tests, and venous blood extraction, all conducted by qualified nursing staff.

An electronic questionnaire was designed in accordance with the recommendations of the International Volcanic Health Hazard Network (IVHHN), an organization under the World Health Organization (WHO), aimed at standardizing epidemiological protocols for assessing health effects in volcanic eruptions [17].

The questionnaire is available on the website of the study (www.estudioisvolcan.com) and included sociodemographic data (age, gender, employment status, occupation type, educational level), variables related to the level of exposure to the volcano (residence before and during the eruption, need for evacuation and subsequent return to the usual residence, access to exclusion zones, involvement in activities related to volcanic ash cleaning, daily hours spent in outdoor environments, and use of masks and eyeglasses for protection), pre-existing comorbidities (lung diseases, cardiovascular diseases, type 2 diabetes, blood hypertension, etc.), acute symptoms (cough, sneezing, wheezing, headache, fatigue, tearing, ocular irritation, etc.), suffering from any respiratory infection (flu, COVID 19 or cold) and visits to emergency services during the eruption, lifestyle factors (smoking habits and leisure-time physical activity). Additionally, the questionnaire included a shortened version of the scale for assessing post-traumatic stress disorder, adapted for the Spanish population [18].

During the visit to the health centre, measurements of weight, height, waist circumference, heart rate, and two separate blood pressure (separately by 10 min) were recorded. Additionally, a venous blood sample of approximately 20 mL was collected, divided into 4 tubes (2 tubes for complete blood count and 2 tubes for biochemistry), for the toxicological determination of persistent contaminants in whole blood and serum.

The tubes for complete blood were stored in a refrigerator at 4 °C, while the biochemistry tubes were centrifuged at 3000 rpm for 10–15 min, and then allowed to rest for 20–25 min until clot retraction. Daily, the samples were transported to the Laboratory of the University Hospital of La Palma and finally stored at the Research Unit of the Hospital Nuestra Señora de Candelaria in Tenerife at -80 °C for the sera and − 20 °C for the whole blood.

In the blood samples, organic contaminants, primarily polycyclic aromatic hydrocarbons (PAHs), will be quantitatively determined due to their possible formation in eruptive processes and their known carcinogenic and teratogenic properties. Among them, the following will be determined: naphthalene, acenaphthene, acenaphthylene, fluorene, anthracene, phenanthrene, pyrene, fluoranthene, benzo(a)anthracene, chrysene, benzo(b)fluoranthene, benzo(k)fluoranthene, benzo(a)pyrene, benzo(ghi)perylene, indene(1,2,3,cd)pyrene, and dibenzo(ah)anthracene. Additionally, inorganic contaminants that may have been emitted in these eruptive processes will be quantified in whole blood. This includes: (a) trace elements (Co, Cr, Cu, Fe, Mn, Ni, Se, and Zn); (b) toxic elements listed in the Agency for Toxic Substances and Disease Registry (ATSDR) inventory, such as Ag, Al, As, Be, Cd, Hg, Pb, Pd, Sb, Sr, Th, Ti, Tl, U, and V; (c) rare earth elements and other minor elements (Au, Bi, Ce, Dy, Eu, Er, Ga, Gd, Ho, In, La, Lu, Nb, Nd, Os, Pr, Pt, Ru, Sm, Sn, Tb, Ta, Tm, Y, and Yb). All these analyses will be performed using gas chromatography coupled with triple quadrupole mass spectrometry (GC-MS/MS) for organic contaminants and inductively coupled plasma mass spectrometry (ICP-MS) for inorganic contaminants. The determinations will be carried out in the Toxicology laboratories of the two public universities of the Canary Islands.

Additionally, each participant underwent forced spirometry to measure lung function following the recommendations by the American Thoracic Society and the European Respiratory Society during the current SARS-CoV-2 pandemic. Forced expiratory volume in the first second (FEV-1) and forced vital capacity (FVC), among other parameters, were measured. Spirometry tests were conducted using a portable spirometer acquired specifically for this study (Sibelmed, model Datospir Touch 3000).

### Statistical analysis

Categorical variables will be presented with their distribution of absolute and relative frequencies. Quantitative variables that follow a normal distribution will be summarized using the mean and standard deviation (± SD), while those that do not follow this distribution will be presented with the median and interquartile range (IQR). To calculate the distance to the volcano, participant home coordinates during the eruption were obtained using the *geodist* command in STATA, and elevation was obtained using the *elevatr* Statistical package in R.

A comparison of the distribution of sociodemographic characteristics, variables related to the level of exposure during the eruption and previous comorbidities of the participants in the general population between the two regions (west and east) was performed. For categorical variables, the Chi-square test were used. Comparisons of means between two regions were performed by Student’s t-test if the variables followed a normal distribution, or by the nonparametric Mann-Whitney U test for asymmetric variables. Finally, multivariate logistic regression models were performed to evaluate the independent effect of the place of residence (west vs. east) on acute symptomatology during the eruption. Those variables considered to be of interest were introduced as adjustment variables. The crude and adjusted odds ratios (OR) are presented together with their 95% confidence intervals (CI). Statistical significance was assumed as *p* < 0.05. Analyses were performed using the statistical package SPSS 26.0® (SPSS Inc., Chicago, IL, USA).

## Results

Preliminary results of the descriptive analysis are presented for the first 1002 participants: 857 participants from the ISVOLCAN cohort, representing the general adult population of La Palma Island, and 145 intervening personnel who accessed the exclusion zone during the eruption.

**Fig. 1:**
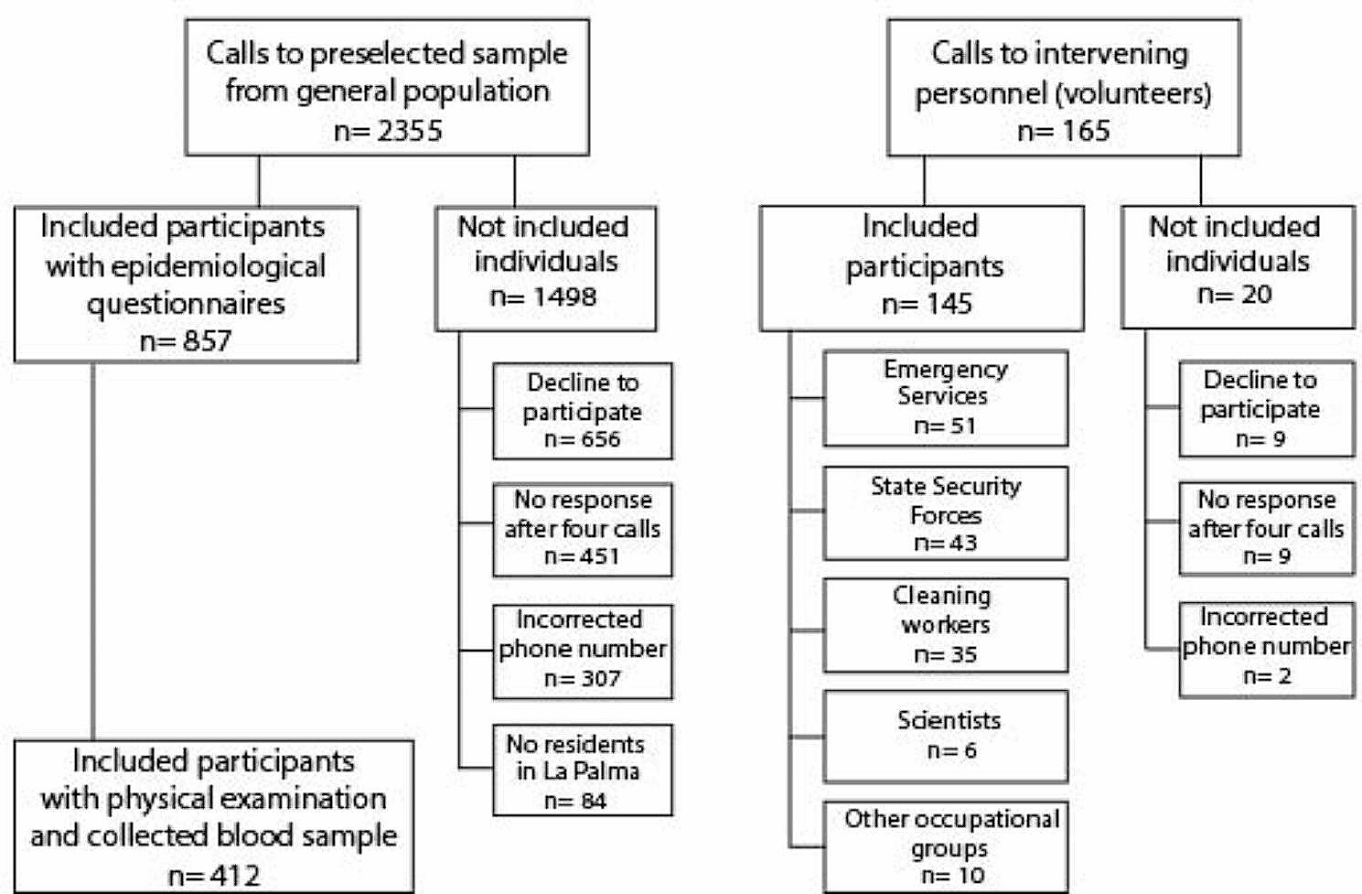
Flowchart of the ISVOLCAN study cohort until December 31, 2022

Figure 1 shows the flowchart of the study sample. As of December 31, 2022, a total of 2355 phone calls were made to randomly selected individuals from the general population, and 857 participants were included (36.4% of those initially selected). In addition to the general population sample, the interveners (*n* = 145) were mainly composed of members of State Security Forces, Emergency Services, and cleaning workers.

Table 1 describes the sociodemographic characteristics of the analysed sample from the general population. The mean age was 50.8 years (± 16.4), with a higher proportion of females. The majority had secondary education, and 20.8% of the sample were unemployed before the eruption; a similar situation was found in the two regions. During the eruption 662 (77.2%) resided in the western region and 198 (22.8%) in the eastern region. The group of participants from the western region presented a higher percentage of women and a higher percentage of unemployed people significantly.

**Table 1:** Sociodemographic characteristics of the general population according to the place of residence during the eruption (western region/eastern region)

	TOTAL (*N* = 857)	WESTERN REGION (*N* = 662)	EASTERN REGION (*N* = 195)		p	
	n (%)	n (%)		n (%)		
**Age (years)** mean (SD)	50.8 (16.4)	50.9 (16.6)		50.4 (15.9)		0.683
**Gender (Female)**	522 (60.9)	415 (62.7)		107 (54.9)		0.049
**Educational level**						
No studies	47 (5.7)	37 (5.8)		10 (5.2)		0.212
Elementary	219 (26.4)	176 (27.5)		43 (22.5)		
Secondary education	361 (43.5)	280 (43.8)		81 (42.4)		
University degree	203 (24.5)	146 (22.8)		57 (29.8)		
**Employment status (before eruption)**						
Retired	154 (18.0)	120 (18.1)		34 (17.4)		0.067
Employee	474 (55.3)	353 (53.3)		121 (62.1)		
Unemployed with benefits	65 (7.6)	54 (8.2)		11 (5.6)		
Unemployed without benefits	113 (13.2)	90 (13.6)		23 (11.8)		
Temporary incapacity	12 (1.4)	9 (1.4)		3 (1.5)		
Permanent incapacity	30 (3.5)	27 (4.1)		3 (1.5)		
Student	9 (1.1)	9 (1.4)		0 (0.0)		
**Employment status (during eruption)**						
Retired	154 (18.0)	120 (18.1)		34 (17.4)		0.047
Employee	458 (53.4)	341 (51.5)		117 (60.0)		
Unemployed with benefits	82 (9.6)	68 (10.3)		14 (7.2)		
Unemployed without benefits	103 (12.0)	82 (12.4)		21 (10.8)		
Temporary incapacity	15 (1.8)	10 (1.5)		5 (2.6)		
Permanent incapacity	36 (4.2)	32 (4.8)		4 (2.1)		
Student	9 (1.1)	9 (1.4)		0 (0.0)		

SD: standard deviation

In the interveners, the mean age was slightly younger (45.7 years (± 11.8)) with a predominance of males (supplemental Table 1).

**Fig. 2:**
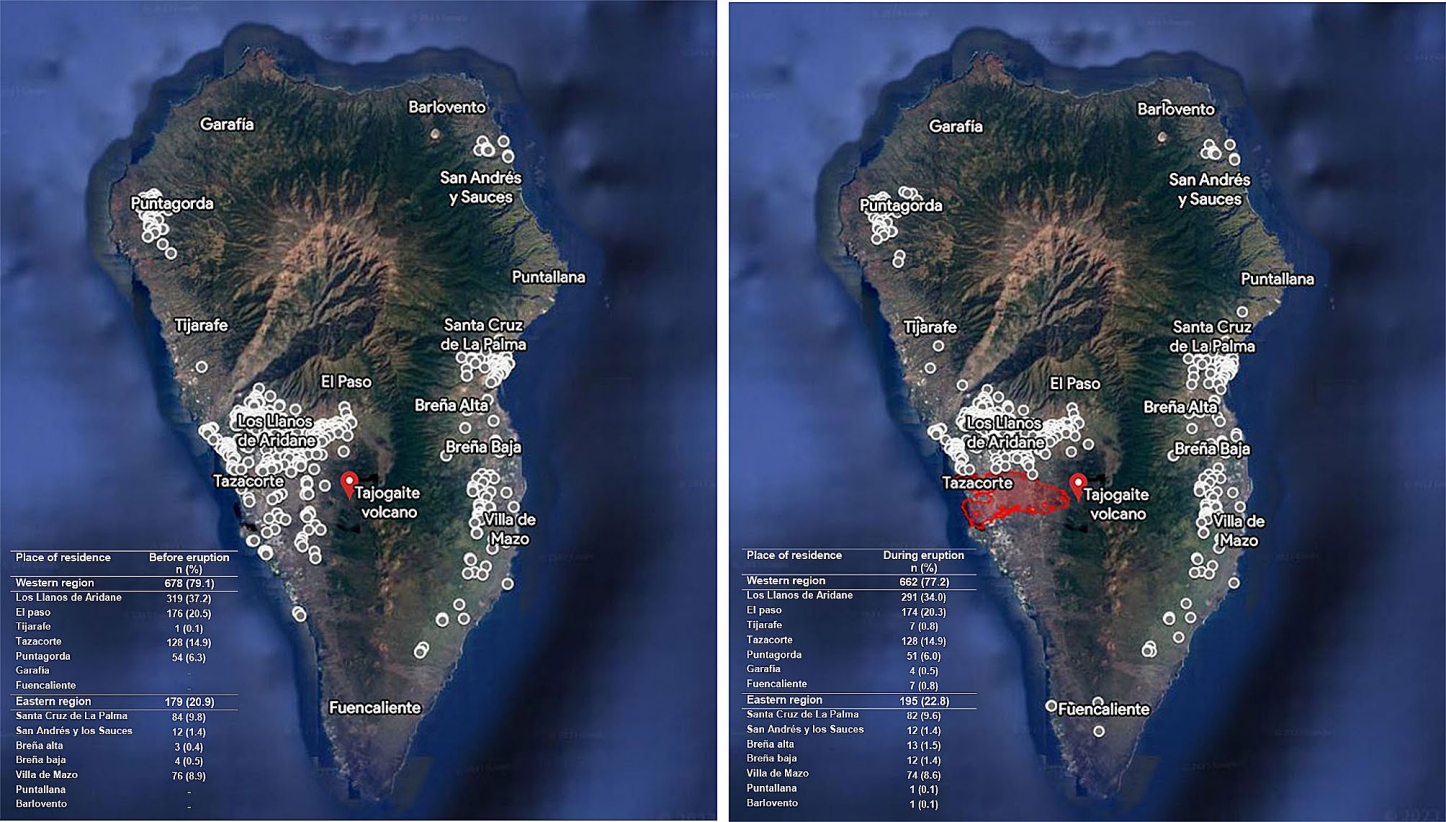
Place of residence of ISVOLCAN cohort participants: (**a**) before and (**b**) during Tajogaite volcano eruption

Figure 2 shows the geolocation of the ISVOLCAN cohort based on the coordinates of participants addresses before and during the eruption in the general population. It can be observed that during the eruption, there was a displacement of residents from the Valle de Aridane area to other parts of the island.

Characteristics related to exposure during the volcanic eruption in general population are described in Table 2. The median distance from participants residence to the volcano during the eruption was 7.1 km (IQR:6.1–9.3); for the western region, it was 6.7 km (IQR: 4.9–7.3), while for the eastern region, it was 10.9 km (IQR: 9.3–12.7). Most of the population in the sample engaged in cleaning up volcanic ash, both inside and outside their homes, using tools with a high capacity for particle projection, such as brooms and blowers. During the eruption, 85% of the general population always used masks when outdoors, with FFP2 masks being the most used. In the bivariate analysis, it found that the location of the usual residence with less distance to the volcano and higher altitude, a more frequent cleaning of volcanic ash both inside and outside the homes and a more daily hours spent in outdoor environments were registered between participants from western region compared to those from the eastern one. The frequency of use of face masks and protective eyeglasses in outdoor environments did not differ between the two regions.

**Table 2:** Aspects related to the level of exposure on the general population according to the place of residence during the eruption (western region/eastern region)

	TOTAL (*N* = 857)	WESTERN REGION(*N* = 662)	EASTERN REGION(*N* = 195)	p
	n (%)	n (%)	n (%)	
**Need for evacuation (usual residence)**	132 (15.4)	116 (17.5)	16 (8.2)	0.002
*Return to usual residence*	46 (34.8)	38 (32.8)	8 (50.0)	0.175
Time to return (days)*	104 (53.5–128)	104 (9.3-129.5)	107.5 (80.5–122.0)	0.839
Distance (usual residence) to volcano (km)*	7.1 (6.1–9.3)	6.7 (4.9–7.3)	10.9 (9.3–12.7)	< 0.001
Altitude in meters (usual residence)*	354.3 (205.4-543.4)	358.8 (320.6-632.8)	269.1 (78.8-415.6)	< 0.001
**Volcanic ash cleaning**	750 (87.5)	590 (89.1)	160 (82.1)	0.009
*Cleaning location*				< 0.001
Outdoor	171 (22.8)	107 (18.1)	64 (40.0)	
Indoor	24 (3.2)	17 (2.9)	7 (4.4)	
Both	555 (74.0)	466 (79,0)	89 (55.6)	
*Cleaning tools*				0.117
High (particle projection)	673 (89.7)	535 (90.7)	138 (86.3)	
Moderate (particle projection)	9 (1.2)	8 (1.4)	1 (0.6)	
Low (particle projection)	68 (9.1)	47 (8.0)	21 (13.1)	
*Cleaning frecuency*				0.004
>=1 once a day	368 (49.9)	302 (52.2)	66 (41.8)	
1–6 times per week	335 (45.5)	257 (44.4)	78 (49.4)	
Every 15 days/monthly	34 (4.6)	20 (3.5)	14 (8.9)	
**Daily hours spent in outdoor environments**				
No or < 1 h	137 (16.1)	99 (15.0)	38 (19.8)	0.010
1–5 h	384 (45.0)	287 (43.4)	97 (50.5)	
> 5 h	332 (38.9)	275 (41.6)	57 (29.7)	
**Frequency of mask use outdoors**				
Always	724 (85.0)	554 (84.2)	170 (87.6)	0.558
Mostly	87 (10.2)	72 (10.9)	15 (7.7)	
Rarely	30 (3.5)	24 (3.6)	6 (3.1)	
Never	11 (1.3)	8 (1.2)	3 (1.5)	
**Mask type**				
Surgical/Hygienic face mask	117 (14.0)	87 (13.4)	30 (15.9)	0.875
FFP2	592 (70.6)	458 (70.6)	134 (70.9)	
FFP3	20 (2.4)	17 (2.6)	3 (1.6)	
Cloth Masks	2 (0.2)	2 (0.3)	0 (0.0)	
Surgical/FFP2 face mask	103 (12.3)	82 (12.6)	21 (11.1)	
Particulate filter mask	4 (0.5)	3 (0.5)	1 (0.5)	
**Frequency of protective eyeglasses use outdoors**				
Always	205 (24.0)	167 (25.4)	38 (19.5)	0.223
Mostly	218 (25.6)	170 (25.8)	48 (24.6)	
Rarely	163 (19.1)	125 (19.0)	38 (19.5)	
Never	267 (31.3)	196 (29.8)	71 (36.4)	

*median and interquartile range (P_25_-P_75_)

The intervining group showed a similar distribution to the general population regarding variables related to volcanic ash cleaning (location, tools used, and cleaning frequency), as well as mask usage frequency and type in outdoor environments (supplemental Table 2).

Table 3 shows baseline characteristics in general population related to lifestyle and pre-existing comorbidities before the eruption and use of healthcare resources and acute symptoms reported by participants during the eruption. The most prevalent pre-eruption comorbidities included blood hypertension (24.3%), depression and anxiety. The most frequently reported acute symptoms by the general population were eye irritation (45.9%), insomnia (44.9%), anxiety and depression (44.7%), and respiratory symptoms. In addition, 12.1% of the sample reported having an emergency visit at a hospital or primary care centres. The main reason for primary care visits was anxiety or depression, while hospital emergency visits were mainly due to osteomuscular traumas. Only 1.8% of the participants reported being hospitalized during the eruption, with surgical intervention being the primary reason. Participants from the western region compared to those from the eastern one were, significantly, more current smokers. Regarding acute symptomatology, western participants showed, in a statistically significant way, higher prevalence of nausea and vomiting, headache, lower respiratory tract symptoms (cough, dyspnea or wheezing), chest pain, insomnia, depression and anxiety, ocular, nasal and ear symptoms.

**Table 3:** Lifestyle factors, prevalence of previous comorbidities, use of healthcare resources and acute symptoms on the general population according to the place of residence during the eruption (western region/eastern region)

	TOTAL (*N* = 857)	WESTERN REGION(*N* = 662)	EASTERN REGION(*N* = 195)	p
	n (%)	n (%)	n (%)	
**LIFESTYLE FACTORS AND PREVIOUS COMORBIDITIES**				
Leisure-time physical activity	480 (56.0)	359 (54.2)	121 (62.1)	0.053
Smoker status				
Never smoked	458 (53.4)	356 (53.8)	102 (52.3)	0.027
Ex-smoker	201 (23.5)	143 (21.6)	58 (29.7)	
Current smoker	198 (23.1)	163 (24.6)	35 (17.9)	
Heart diseases	71 (8.3)	56 (8.5)	15 (7.7)	0.733
Asthma	71 (8.3)	61 (9.2)	10 (5.1)	0.069
COPD/Chronic bronchitis	21 (2.5)	20 (3.0)	1 (0.5)	0.061
Blood hypertension	208 (24.3)	157 (23.7)	51 (26.2)	0.485
Type 2 diabetes mellitus	82 (9.6)	62 (9.4)	20 (10.3)	0.710
Dyslipemia	83 (9.7)	63 (9.5)	20 (10.3)	0.759
Depression/anxiety	108 (12.6)	89 (13.4)	19 (9.7)	0.171
Cancer	33 (3.9)	26 (3.9)	7 (3.6)	0.829
**ACUTE SYMPTOMS AND USE OF HEALTHCARE RESOURCES**				
Nausea/vomiting	62 (7.2)	55 (8.3)	7 (3.6)	0.025
Headache	254 (29.6)	212 (32.0)	42 (21.5)	0.005
Cough	221 (25.8)	187 (28.2)	34 (17.4)	0.002
Dyspnea	180 (21.0)	150 (22.7)	30 (15.4)	0.028
Wheezing	63 (7.4)	55 (8.3)	8 (4.1)	0.048
Lower respiratory tract	312 (36.4)	264 (39.9)	48 (24.6)	< 0.001
Chest pain	47 (5.5)	44 (6.6)	3 (1.5)	0.006
Accidents	10 (1.2)	7 (1.1)	3 (1.5)	0.703
Insomnia	385 (44.9)	334 (50.5)	51 (26.2)	< 0.001
Depression/anxiety	383 (44.7)	329 (49.7)	54 (27.7)	< 0.001
Nasal/ear	288 (33.6)	240 (36.3)	48 (24.6)	0.002
Ocular	393 (45.9)	316 (47.7)	77 (39.5)	0.042
Muscle pain	101 (11.8)	81 (12.2)	20 (10.3)	0.451
General malaise	18 (2.1)	15 (2.3)	3 (1.5)	0.777
Cutaneous	42 (4.9)	31 (4.7)	11 (5.6)	0.586
Digestive	1 (0.1)	1 (0.2)	0 (0.0)	1.000
Pharyngeal	45 (5.3)	30 (4.5)	15 (7.7)	0.082
**Emergency room visit**	104 (12.1)	84 (12.7)	20 (10.3)	0.361
Family Physician	45 (5.3)	37 (5.6)	8 (4.1)	0.413
Primary care emergency department	48 (5.6)	39 (5.9)	9 (4.6)	0.496
Hospital emergency department	23 (2.7)	14 (2.1)	9 (4.6)	0.058
**Hospital admission**	15 (1.8)	11 (1.7)	4 (2.1)	0.756
**Respiratory infection during eruption**	81 (9.5)	67 (10.1)	14 (7.2)	0.217
COVID 19	36 (4.2)	29 (4.4)	7 (3.6)	0.628
Influenza	15 (1.8)	14 (2.1)	1 (0.5)	0.212
Cold	33 (3.9)	27 (4.1)	6 (3.1)	0.523

COPD: chronic obstructive pulmonary disease

In the interveners, the acute symptoms reported during the eruption were like those of the general population, as well as the utilization of healthcare services. However, the percentage of hospitalizations was lower in this group (supplemental Table 3).

Table 4 shows the adjusted and unadjusted effect of region of residence during the volcano eruption (west/east) in general population on each of the most prevalent acute symptoms that showed statistically significant differences between the two regions. Age, gender, education level, employment, distance to the volcano, ash cleaning, type of cleaning tool used, daily hours in outdoor environments and type of smoker were entered as adjustment variables in all multivariate models. In addition, for the acute symptom lower respiratory tract symptoms, we adjusted for having suffered from any respiratory infection (influenza, COVID 19 or cold) during the months of the volcano eruption. Adjusted multivariate analysis results show that participants in the western region had a higher likelihood of lower respiratory tract symptoms (OR 1.99; 95% CI:1.33–2.99), depression and anxiety (OR 1.95; 95% CI:1.30–2.93) and insomnia (OR 2.03; 95% CI:1.33–3.09), compared to those in the eastern region.

**Table 4:** Association of place of residence on the general population (western vs. eastern region) in acute symptomatology during the eruption of the volcano. Logistic regression

Acute symptoms	OR (CI 95%) (western vs. east region)	p	OR_a_ (CI 95%) (western vs. east region)	p
Lower respiratory tract (Cough, Dyspnea, Wheezing)	2.03 (1.42–2.92)	< 0.001	1.99 (1.33–2.99)*	0.001
Depression/anxiety	2.58 (1.82–3.66)	< 0.001	1.95 (1.30–2.93)**	0.001
Insomnia	2.88 (2.02–4.10)	< 0.001	2.03 (1.33–3.09)**	0.001
Ocular	1.40 (1.01–1.94)	0.043	1.33 (0.91–1.96)**	0.140
Headache	1.72 (1.18–2.51)	0.005	1.26 (0.80–1.97)**	0.321
Nasal/ear	1.74 (1.21–2.50)	0.003	1.51 (0.99–2.28)**	0.053

OR: odds ratio; OR_a_: adjusted odds ratio; CI: confidence interval

*Multivariate logistic regression adjusted for age, gender, education level, employment, distance to the volcano, ash cleaning, type of cleaning tool used, daily hours in outdoor environments and type of smoker and suffering from any respiratory infection during the eruption

**Multivariate logistic regression adjusted for age, gender, education level, employment, distance to the volcano, ash cleaning, type of cleaning tool used, daily hours in outdoor environments and type of smoker

## Discussion

This article presents the methodology of the ISVOLCAN study, as well as a descriptive analysis of the baseline characteristics of the first 1002 participants (857 participants from the general adult population of La Palma Island, and 145 interveners, potentially highly exposed).

After the initial telephone contact was established with the selected individuals from the general population of the island, an initial response rate of 36.4% was observed. Although a higher participation rate was expected, the conditions of uncertainty and vulnerability experienced by the population immediately after the eruption was extinguished and during the subsequent months, generated certain limitations. At the beginning of the ISVOLCAN study, part of the evacuated population was still displaced or involved in bureaucratic and administrative procedures related to the disaster.

As mentioned previously, epidemiological data for each participant were collected through a health questionnaire. Analysis of this data revealed that the participants had a mean age within the working-age range, with a predominance of women and most individuals who had completed secondary education. The recruited population mainly resided in the municipalities affected by the volcano, with the highest number of displacements during the eruption occurring among the inhabitants of Los Llanos de Aridane, which coincided with the movement of the lava flows. Regarding the intervining group, it was observed that they were younger and predominantly male, reflecting the male dominance in certain professions related to the field of public safety.

Factors related to the level of exposure of the participants were also considered in the analysis. It was observed that the proximity to the volcano was about 7 km, even less for the residents of Valle de Aridane. This proximity is unusual compared to other volcanic phenomena documented in scientific literature. For instance, in the case of Holuhraun, population centres were located at least 100 km away from the volcano, with only a few isolated farms found at a closer distance, approximately 70 km [19]. Another recent example concerns the Nyragongo or Nyamulagira volcanoes in the Republic of Congo, which affected a population of nearly one million people around the volcano, at approximately 15–30 km [20]. Therefore, in La Palma Island, the local population resided much closer to the eruption at the time compared to other mentioned populations.

Various health risks associated with the size of PM and their potential environmental impact on agriculture and water reservoirs have been reported [4]. Indeed, the deposition of several heavy metals, such as chromium and arsenic, in soils near volcanic eruptions has been documented, both of which have carcinogenic effects at certain levels [19]. In line with this, a very recent publication shows the chemical characterization of ash samples from Tajogaite eruption, founding that the most of the water-soluble compounds were SO_4_, F, Cl, Na, Ca, Ba, Mg and Zn; worryingly, the authors conclude that F and Cl concentration may exceed both the recommended levels for irrigation purpose and for health [21].

Moreover, the size of PM is of critical importance; particles smaller than 10 μm (PM_10_) can penetrate and reach the alveolar region of the lungs [3], while those smaller than 2.5 μm (PM_2.5_) may even cross the lung barrier and enter the bloodstream. There is an extensive body of evidence in relation to the health effects of the long-term exposure to PM 2.5 or lesser. The main reported effects are on all-causes and cause-specific mortality [22], incidence of cardiovascular or respiratory diseases [19, 23], incidence of endocrine and metabolic disorders such as type 2 diabetes [24] and incidence of lung cancer among others, even at concentrations below current EU limit values and possibly WHO Air Quality Guidelines [25]. However, to the best of our knowledge, there are no published studies that analyze the potential effects of degassing exposure on the population of La Palma, neither before nor during the eruption.

During the Tajogaite eruption, daily air quality monitoring was carried out through eight stations located in different points of Valle de Aridane and the eastern region of the island. Based on these records, the average levels of SO_2_ concentration in the island were recently published, and it was observed that the threshold recommended as safe by the European Commission was exceeded in the Valle area during 1 to 4% of the eruption duration. Furthermore, during the first month of the eruption, the threshold of 400 μm-3 was frequently exceeded, especially in the later stages of the phenomenon, in contrast to the emissions of particulate matter [2, 26].

It is noteworthy to mention that, due to the recommendations of authorities and scientists, as well as the activation of volcanic emergency protocols, the integrity of the population was successfully safeguarded. However, it is reasonable to assume that the displacements of the evacuated population during the eruption could have had an impact on their health. Throughout the volcanic event, the island’s population received daily information about the necessary preventive measures in each municipality, based on air quality and the evolution of volcanic ash. In the case of our sample from the general population, 15.4% were evacuated during the eruption, and less than half of the evacuated individuals returned to their usual homes after an average of approximately 3 months.

On the other hand, exposure to volcanic gases and ash has been widely associated with increased respiratory morbidity and short-term irritation in the respiratory tract, ocular mucosa, and skin due to their chemical and mechanical irritant effects [3, 19, 27]. In the case of ISVOLCAN cohort participants, ocular and upper respiratory tract irritation were the most frequent acute symptoms. These findings are consistent with epidemiological studies conducted in the general population, both during the acute phase [28] and 6–9 months after exposure [11], as well as in highly exposed professionals [29]. Other studies evaluating the reasons and number of visits to hospital emergency departments have detected an increase in visits due to respiratory diseases and ocular disorders [30, 31].

During the volcanic eruption, a significant proportion of the participants carried out ash cleaning tasks both indoors and outdoors, thereby increasing their exposure to the emitted material. As the eruption coincided with the second year of the SARS-CoV-2 pandemic, the population already had access to masks and was used to wearing them; the majority of the participants stated using masks when outdoors, with FPP2 masks being the most commonly used in these environments during the eruption, as they have demonstrated effectiveness in protecting against the inhalation of volcanic ash [32]. Certainly, it is imperative to maintain a surveillance over this excessive exposure in the coming years to comprehensively gauge potential medium and long-term repercussions. In the aftermath of the Tajogaite volcanic eruption, numerous supplementary investigations have been instigated, in addition to ISVOLCAN, with the aim of enhancing the monitoring of the health of the local population. Notably, the ASHES study is among these initiatives, with its principal focus being the assessment of respiratory health outcomes associated with exposure to volcanic emissions [33].

Moreover, prior investigations following volcanic eruptions have demonstrated a notable rise in the occurrence of psychiatric disorders within the general population [34]. Evacuated individuals, in particular, exhibited a pronounced prevalence of post-traumatic stress and depressive symptoms [35]. During the eruption period, nearly half of the individuals reported insomnia and symptoms indicative of mood disorders, such as anxiety or depression. Notably, those who had to undergo evacuation displayed a higher incidence of these symptoms. The eruption caused significant disruptions in the daily routines of the population in specific municipalities, especially those directly affected by evacuation orders.

The elevated prevalence of anxiety and depression can be related to several factors, including increased work demands during the eruption and the uncertainty concerning personal health, the well-being of others, property, and crop security, as well as the outlook for the future. Furthermore, given the substantial number of seismic events and the explosive nature of the eruption, it is plausible that these anxiety-related symptoms contributed to the substantial percentage of reported insomnia among the affected population.

Adjusted multivariate analysis results show that participants in the western region compared to those in the eastern region had a higher likelihood of lower respiratory tract symptoms, depression and anxiety, and insomnia. These results are similar to those found in the few epidemiological studies conducted in the general population that evaluate symptomatology, acute or short-term, during the eruption according to the level of exposure. These results are in concordance to previous evidence [11, 36].

Furthermore, the recognition of volcanic eruptions as sources of toxic elements underscores the environmental exposure faced by populations residing in close proximity to these emission sites. Environmental studies conducted worldwide, including the Canary Islands, have consistently identified volcanic eruptions as significant contributors of inorganic elements known to be toxic to humans, such as Se, Cd, Pb and Hg [37, 38]. Notably, recent findings from the ISVOLCAN study have documented elevated levels of Fe, Al, Ti, V, Ba, Pb, Mo, Co, and Rare Earths in banana crops on the island during the eruption period [39].

However, studies focused on monitoring toxin levels in populations affected by eruptions are limited, primarily due to the challenge of simultaneously quantifying these inorganic toxins in blood samples collected from affected individuals. Furthermore, the necessary analytical methods are mostly expensive, limiting their inclusion into epidemiological studies. In this context, our research team, as experts in toxicological analysis of both major inorganic and organic pollutants, is presently conducting determinations using venous blood samples from study participants, although results are pending.

The main limitation of the ISVOLCAN study, as is common in cohort studies, is its high cost, which is exacerbated in our case by logistical difficulties inherent in a fragmented territory like the Canary Islands, limiting the transfer of biological samples and human or material resources between islands. Additionally, while the participants were randomly selected from the general population, there may exist a selection bias if those who chose not to participate had some differential characteristics (e.g., older age, pre-existing health issues, etc.) compared to the participants, which could limit the detection of certain relevant associations.

Furthermore, the epidemiological data relies on self-reporting by the participants, which could introduce information biases affecting the validity of the results. Additionally, the high percentage of losses during follow-up, related to this type of design, could generate a survival bias. To address these concerns, several methodological strategies have been implemented. The sample size was increased to more than double the initial estimate, that is why recruitment and inclusion of participants are ongoing at this moment. Moreover, as a strength of the study, data collection started as soon as possible after the eruption was finished, carried out by personnel specially trained to ensure rigor and thoroughness in the process, following the recommendations of the IVHHN regarding epidemiological data records for such phenomena. Additionally, prior to analysis, the data undergo rigorous quality control and verification processes.

Given that the data come from a randomized sample of the general population of the island, followed over several years, this study will allow for the detection of causal associations. It is worth noting that the inclusion of interveners in the ISVOLCAN cohort provides a significant area of study since they can be considered as individuals with high prior exposure.

## Conclusion

The ISVOLCAN study has been meticulously designed as a 10-year follow-up study aimed at assessing the medium to long-term health impact on the adult general population of La Palma Island following the recent eruption of the Tajogaite volcano. Despite currently being in a recruitment phase, the study has successfully completed several stages of biological sample collection and biomedical data gathering. Once the baseline measurements are finalized and toxicological determinations are conducted, data from over 2000 individuals with varying levels of exposure during the eruption are expected to be obtained. Lastly, in our knowledge this study is the first to publish data related to the short-term health impact on the population of La Palma following the eruption of the Tajogaite volcano.

### Electronic supplementary material

Below is the link to the electronic supplementary material.

Supplementary Material 1

## Data Availability

The data that support the findings of this study are not openly available due to reasons of sensitivity and are available from the corresponding author upon reasonable request.

## Abbreviations

CO: Carbon monoxide
CO_2_: Carbon dioxide
SO_2_: Sulfur dioxide
HCl: Hydrogen chloride
HF: Hydrogen fluoride
H_2_S: Hydrogen sulfide
PM: Particulate matter
IVHHN: International Volcanic Health Hazard Network
WHO: World Health Organization
PAHs: Primarily polycyclic aromatic hydrocarbons
ATSDR: Agency for Toxic Substances and Disease Registry
SD: Standard deviation
IQR: Interquartile range
PM_2.5_: Particulate matter ≤ 2.5 μm
PM_10_: Particulate matter < 10 μm
