# Supplementary material for: Health impact of the Tajogaite volcano eruption in La Palma population (ISVOLCAN study): rationale, design, and preliminary results from the first 1002 participants
Source: Environ Health. 2024 Feb 13;23:19. doi: 10.1186/s12940-024-01056-4 (PMC10863256; doi:10.1186/s12940-024-01056-4)
Supplement: Supplementary file 1 — Supplementary Material 1 [file 12940_2024_1056_MOESM1_ESM.docx]

**SUPPLEMENTARY MATERIAL**

| Supplemental Table 1. Sociodemographic characteristics on intervining personnel. | |
| --- | --- |
|  | **INTERVINING PERSONNEL**  **(N=145)** |
|  | **n (%)** |
| **Age (years)** mean (SD) | 45.7 (11.8) |
| **Gender (Female)** | 25 (17.2) |
| **Educational level** |  |
| No studies | 1 (0.7) |
| Elementary | 20 (13.9) |
| Secondary education | 96 (66.7) |
| University degree | 27 (18.8) |
| **Employment status (before eruption)** |  |
| Retired | 9 (6.2) |
| Employee | 118 (81.4) |
| Unemployed with benefits | 7 (4.8) |
| Unemployed without benefits | 9 (6.2) |
| Temporary incapacity | 2 (1.4) |
| Permanent incapacity | 0 (0.0) |
| Student | 0 (0.0) |
| **Place of residence (before eruption)** |  |
| ***La Palma (Western region)*** | **102 (70.3)** |
| Los Llanos de Aridane | 43 (29.7) |
| El paso | 43 (29.7) |
| Tijarafe | 2 (1.4) |
| Tazacorte | 9 (6.2) |
| Puntagorda | 5 (3.4) |
| Garafía | - |
| Fuencaliente | - |
| ***La Palma (Eastern region)*** | **26 (17.9)** |
| Santa Cruz de La Palma | 8 (5.5) |
| San Andrés y los Sauces | 1 (0.7) |
| Breña alta | 4 (2.8) |
| Breña baja | 4 (2.8) |
| Villa de Mazo | 8 (5.5) |
| Puntallana | 1 (0.7) |
| Barlovento | - |
| ***Other canarian islands*** | **17 (11.7)** |
| El Hierro | 2 (11.8) |
| Lanzarote | 3 (17.6) |
| Tenerife | 12 (70.6) |

| Supplemental table 2. Aspect related to the level of exposure on intervining personnel. | |
| --- | --- |
|  | **INTERVINING PERSONNEL (N=145)** |
|  | **n (%)** |
| **Need for evacuation (usual residence)** | 16 (11.0) |
| *Return to usual residence* | 7 (43.8) |
| *Time to return (days)** | 49 (2-111) |
| **Distance (usual residence) to volcano (km)*** | 7.0 (4.9-11.9) |
| **Altitude in meters (usual residence)*** | 374.8 (305.3-577.8) |
| **Access to exclusion zones** | 137 (94.5) |
| **Volcanic ash cleaning** | 122 (84.1) |
| *Cleaning location* |  |
| Outdoor | 44 (36.1) |
| Indoor | 2 (1.6) |
| Both | 76 (62.3) |
| *Cleaning tools* |  |
| High (particle projection) | 112 (91.8) |
| Moderate (particle projection) | 3 (2.5) |
| Low (particle projection) | 7 (5.7) |
| *Cleaning frecuency* |  |
| >=1 once a day | 63 (52.5) |
| 1-6 times per week | 49 (40.8) |
| Every 15 days/monthly | 8 (6.7) |
| **Daily hours spent in outdoor environments** |  |
| No or <1 hour | 5 (3.4) |
| 1-5 hours | 15 (10.3) |
| >5 hours | 125 (86.2) |
| **Frequency of mask use outdoors** |  |
| Always | 121 (84.6) |
| Mostly | 14 (9.8) |
| Rarely | 3 (2.1) |
| Never | 5 (3.5) |
| **Mask type** |  |
| Surgical/Hygienic face mask | 5 (3.6) |
| FFP2 | 91 (66.4) |
| FFP3 | 25 (18.2) |
| Cloth Masks | 0 (0.0) |
| Surgical/FFP2 face mask | 10 (7.3) |
| Particulate filter mask | 6 (4.4) |
| **Frequency of protective eyeglasses use outdoors** |  |
| Always | 59 (41.8) |
| Mostly | 46 (32.6) |
| Rarely | 17 (12.1) |
| Never | 19 (13.5) |
| *median and interquartile range (P_25_-P_75_) | |

| Supplemental Table 3. Lifestyle factors, prevalence of previous comorbidities, use of healthcare resources and acute symptoms during the eruption on the intervining personnel. | |
| --- | --- |
|  | **INTERVINING PERSONNEL**  **(N=145)** |
|  | **n (%)** |
| **LIFESTYLE FACTORS AND PREVIOUS COMORBIDITIES** | |
| Leisure-time physical activity | 100 (69.0) |
| Smoker status |  |
| Never smoked | 78 (53.8) |
| Ex-smoker | 25 (17.2) |
| Current smoker | 42 (29.0) |
| Heart diseases | 7 (4.8) |
| Asthma | 16 (11.0) |
| COPD/Chronic bronchitis | 1 (0.7) |
| Blood hypertension | 29 (20.0) |
| Type 2 diabetes mellitus | 7 (4.8) |
| Dyslipemia | 11 (7.6) |
| Depression/anxiety | 5 (3.4) |
| Cancer | 2 (1.4) |
| **ACUTE SYMPTOMS AND USE OF HEALTHCARE RESOURCES** | |
| Nausea/vomiting | 14 (9.7) |
| Headache | 38 (26.2) |
| Cough | 37 (25.5) |
| Dyspnea | 22 (15.2) |
| Wheezing | 17 (11.7) |
| Chest pain | 6 (4.1) |
| Accidents | 2 (1.4) |
| Insomnia | 40 (27.6) |
| Depression/anxiety | 47 (32.4) |
| Nasal/ear | 47 (32.4) |
| Ocular | 55 (37.9) |
| Muscle pain | 11 (7.6) |
| General malaise | 5 (3.4) |
| Cutaneous | 3 (2.1) |
| Digestive | 1 (0.7) |
| Pharyngeal | 4 (2.8) |
| **Emergency room visit** | 13 (9.0) |
| Family Physician | 2 (1.4) |
| Primary care emergency department | 8 (5.5) |
| Hospital emergency department | 3 (2.1) |
| **Hospital admission** | 1 (0.7) |
| COPD: chronic obstructive pulmonary disease. | |
